# Supplementary material for: The impact of anthropogenic inputs on lithium content in river and tap water
Source: Nat Commun. 2019 Dec 3;10:5371. doi: 10.1038/s41467-019-13376-y (PMC6890772; doi:10.1038/s41467-019-13376-y)
Supplement: Supplementary file 1 — Supplementary Information [file 41467_2019_13376_MOESM1_ESM.pdf]

Supplementary Information for

The impact of anthropogenic inputs on lithium content in river and tap water

by Choi *et al*.

Supplementary Figures

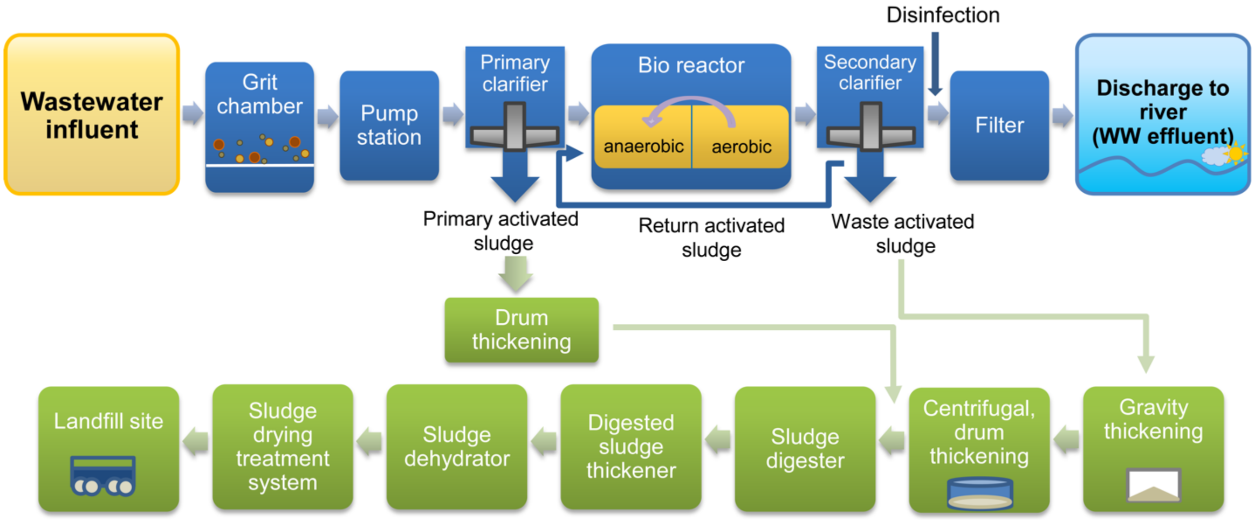

Supplementary Figure 1. Schematic diagram showing the wastewater (WW) treatment processes, and the relationship between treated waters (WW effluent) and the Han River in Seoul (modified from Seoul Tancheon Water Reclamation Centers; http://www.tancheon.com/kr/). Grit chamber removes solid phases in wastewater. Primary clarifier removes the pollutants by gravity during 3 hours. Bioreactor decomposes nutrients and organic matter. Secondary clarifier removes organic matter by gravity.

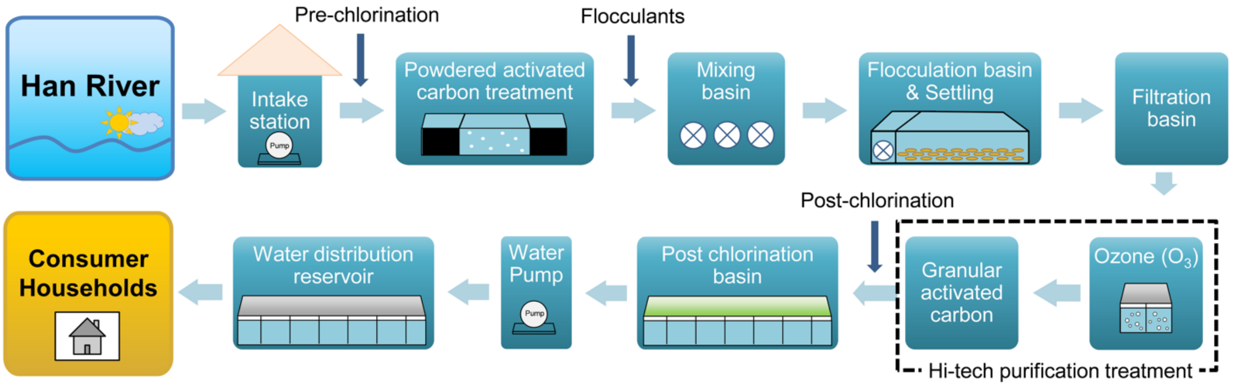

Supplementary Figure 2. Schematic diagram showing the tap water purification processes from water extracted from the Han River reservoir downstream the Paldang Dam (modified from the Office of Waterworks Seoul Metropolitan Government; https://arisu.seoul.go.kr/index.jsp).

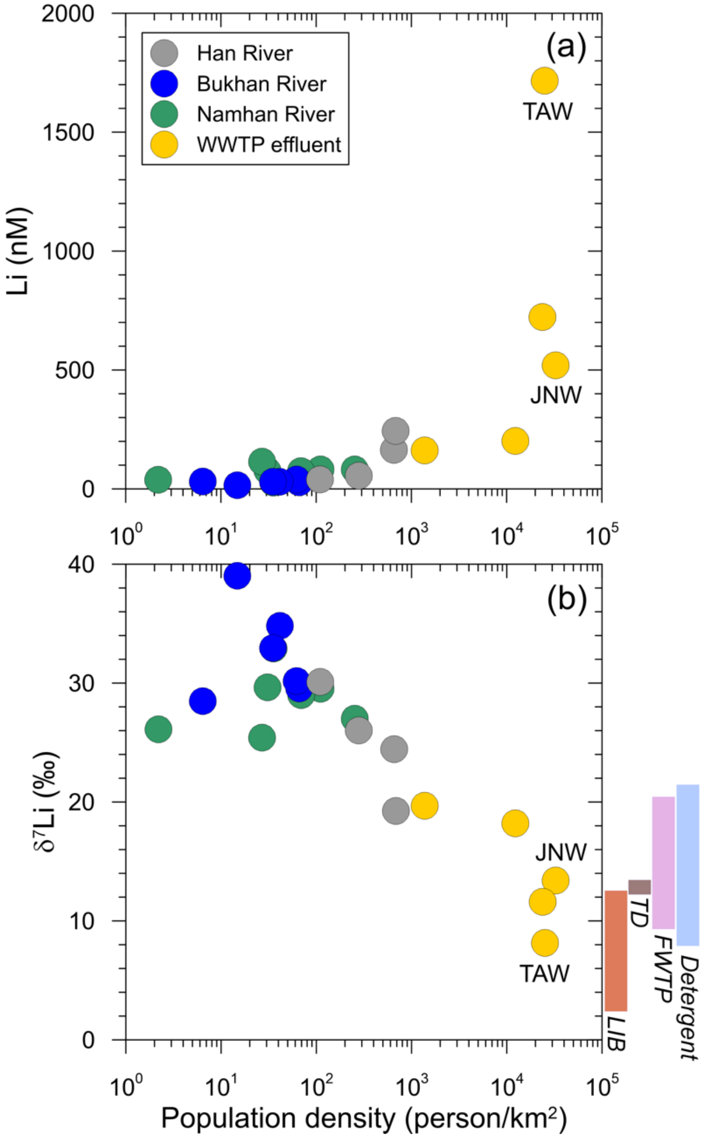

Supplementary Figure 3. Lithium concentrations (a) and Li isotope compositions (b) in river waters and wastewaters, as a function of the population density. LIB, TD and FWTP represent secondary Li-ion battery, therapeutic drug and food waste treatment plant, respectively. The low δ^7Li values measured in the wastewater (yellow circle) are compatible with those of the anthropogenic sources, and particularly the Li-rich materials (LIB and TD) (see text and Supplementary Table 5). Wastewater effluent coming from the "TAW" WWTP is strongly enriched in Li compared to the others. The high Li content associated to low δ^7Li value is likely due to the food waste treatment plant located closely.

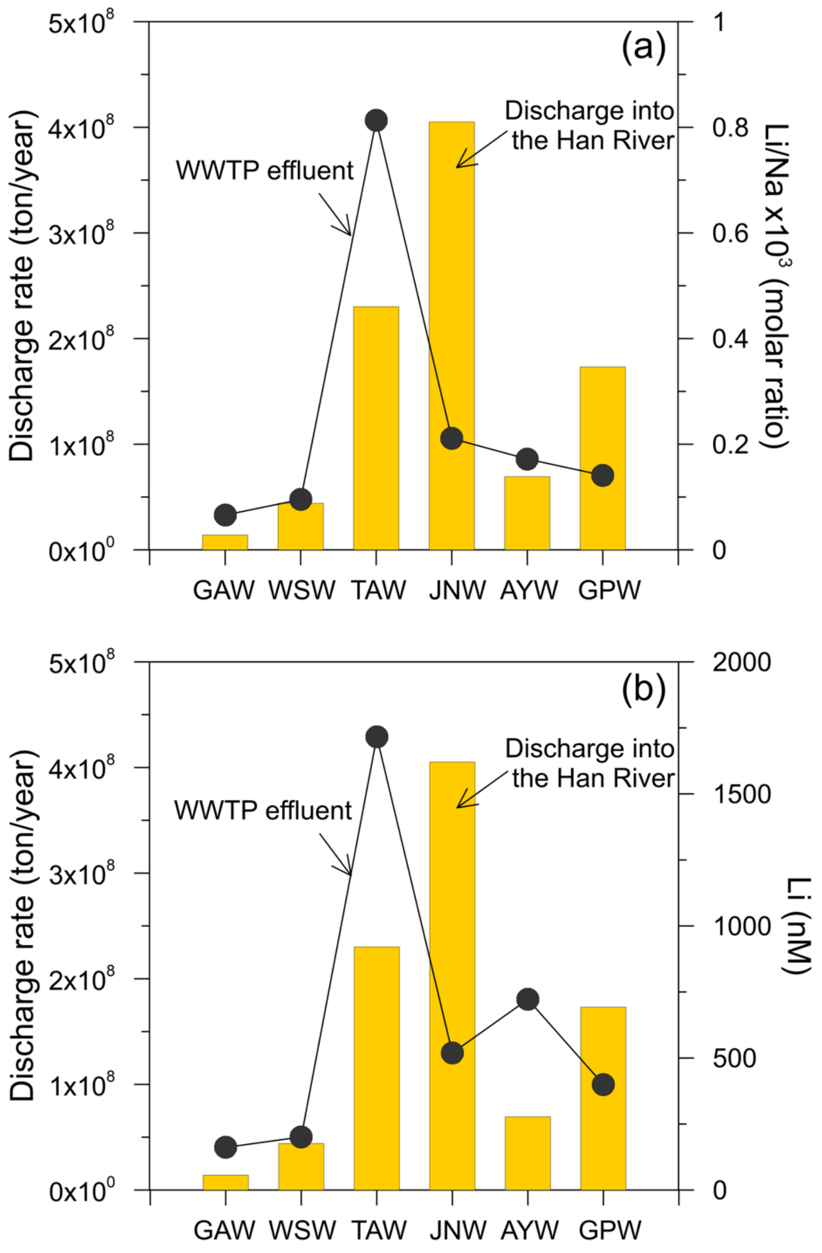

Supplementary Figure 4. Relationship between the discharge rate of WWTP (yellow bar) and Li/Na ratio (a), and Li concentration (b) in the WW effluent. The highest Li content and Li/Na ratio are measured for the "TAW" WWTP and is best explained by the Li-rich wastewaters coming from the only food waste treatment plant of Seoul (Fig. 1B). Indeed, we have shown that the food wastewater contains high Li content (2.90 ± 0.45 μM, 1σ, n=2; Supplementary Table 3). The discharge rate of TAW is much lower than that of JNW, explaining the discrepancy with the population density observed for these samples (Supplementary Figure 3).

Supplementary Tables

Supplementary Table 1

Geographical information of the Han River Basin[a].

Sample	Land use[b]							Population[c]
	Forest	Agricultural area	River	Resident	Field	Golf	Industrial district	
	(%)							(person)
Han River (HR)								
HR1	79.8	13.6	2.5	2.5	1.2	0.2	0.3	2,063,811
HR2	76.7	13.3	2.5	3.1	1.2	0.2	3.1	5,458,771
HR3	77.6	13.4	2.6	4.6	1.3	0.2	0.4	13,216,728
HR4	77.3	13.4	2.6	4.8	1.3	0.2	0.4	13,915,532
Bukhan River (BR)								
BR1	86.2	8.2	2.8	2.0	0.8	0.0	0.1	469,793
BR2	87.4	6.7	3.1	2.0	0.7	0.0	0.0	339,494
BR3	90.1	5.2	2.8	1.1	0.8	0.0	0.0	41,682
BR4	84.9	12.1	1.1	1.3	0.6	0.0	0.0	35,928
BR5	88.5	5.8	3.1	2.0	0.5	0.0	0.0	33,830
BR6	90.0	5.8	1.0	1.6	1.5	0.0	0.0	13,372
Namhan River (NR)								
NR1	76.3	17.2	2.2	2.5	1.4	0.2	0.2	1,182,930
NR2	75.1	17.6	1.4	3.9	1.4	0.4	0.3	328,185
NR3	80.1	14.4	2.0	1.9	1.3	0.1	0.1	539,388
NR4	84.6	11.8	1.1	1.2	1.3	0.1	0.1	148,762
NR5	86.5	10.0	1.0	1.1	1.3	0.1	0.0	68,086
NR6	81.4	14.7	1.2	1.3	1.2	0.1	0.1	55,302
NR7	97.0	1.2	0.8	0.9	0.1	0.0	0.0	75

[a] data from a ArcGIS 10 (Esri, Redlands, CA, USA)

[b] Land use data from Environmental geographic information service, EGIS (https://egis.me.go.kr)

[c] Population data from biz-gis (www.biz-gis.com)

Supplementary Table 2

Elemental and Li isotope geochemistry of river water samples.

Sample	T	pH	EC	Ca	Mg	Na	K	Si	Sr	Cl	SO_4	HCO_3[a]	NO_3	CBE[b]	Li	δ^7Li
	(°C)		(μS/cm)	(μM)										(%)	(nM)	(‰)
Han River (HR)																
HR1	25.1	7.1	135	357	116	285	58.1	92.7	0.90	276	98.0	657	137	0.9	39.6	30.1
HR2	25.8	7.4	152	391	131	351	65.1	81.5	1.00	336	111	739	130	1.2	55.7	26.0
HR3	26.9	7.2	181	455	149	443	81.8	76.9	1.08	439	130	860	141	0.9	164	24.4
HR4	26.4	7.0	243	523	162	725	137	92.3	1.22	703	146	934	245	1.3	244	19.2
Bukhan River (BR)																
BR1	23.6	7.0	96.8	258	70.0	199	37.4	173	0.68	166	78.5	351	197	1.2	27.8	29.5
BR2	23.6	7.7	178	553	159	346	61.2	153	1.25	325	97.8	867	201	7.1	40.0	30.2
BR3	18.4	7.4	100	293	82.4	198	35.2	94.6	0.83	160	81.3	470	135	3.0	15.9	39.0
BR4	21.3	7.5	144	405	125	257	50.9	178	1.26	281	95.1	507	337	2.0	30.2	34.8
BR5	27.4	8.1	105	315	110	184	37.6	51.4	0.71	142	63.4	617	93.8	4.4	29.9	32.9
BR6	20.4	7.5	76.0	170	57.9	177	24.8	157	0.53	166	48.9	271	116	0.6	31.0	28.5
Namhan River (NR)																
NR1	29.1	7.6	189	475	138	439	95.7	138	1.33	431	148	844	161	0.9	81.3	29.5
NR2	24.9	7.6	179	509	122	407	77.9	173	1.48	415	106	834	222	1.9	81.6	27.0
NR3	26.2	7.5	188	542	173	414	88.7	137	1.18	397	130	1031	104	3.8	74.9	29.0
NR4	26.1	8.1	250	900	261	217	39.9	115	1.64	285	167	1593	224	2.8	75.2	29.6
NR5	25.5	8.2	254	974	242	202	32.0	87.5	2.22	292	212	1671	184	1.8	114	25.4
NR6	25.7	8.0	241	803	276	259	48.1	137	1.18	356	128	1476	283	1.9	26.7	32.9
NR7	20.4	7.6	44.2	114	38.9	111	17.2	129	0.24	64.1	35.1	197	99.2	0.3	37.7	26.1

[a] HCO_3 ≈ Total alkalinity

[b] Charge Balance Error (CBE) = $(TZ^+ - TZ^-)/(TZ^+ + TZ^-) \times 100$

Supplementary Table 3

Elemental and Li isotope geochemistry of wastewaters from the Seoul treatment plants (WWTP).

Sample[a]	Drainage area[b]	Population[b]	Population density[c]	Discharge rate[b]	T	pH	EC	Ca	Mg	Na	K	Sr	Cl	SO_4	HCO_3[d]	NO_3	Li	δ^7Li	$1\sigma_m$[e]	n
	km^2	($\times 10^3$)	(person/km^2)	($\times 10^6$ ton/year)	(°C)		(μS/cm)	(μM)									(nM)	(‰)		
Influent of WWTP																				
GAW-I	32	44	1,375	14.1	24.1	7.2	648	920	300	1990	340	1.71	1930	390	3530	n.d.	124	21.7	-	-
WSW-I	27	337	12,325	44.1	25.7	7.2	623	880	270	2350	350	1.51	2160	360	2980	n.d.	214	19.4	-	-
TAW-I	71	1,776	25,185	230	26.7	7.1	739	820	350	2780	420	1.54	2600	300	3610	n.d.	1586	7.7	-	-
JNW-I	112	3,641	32,580	405	26.8	7.1	690	750	250	2640	400	1.40	2170	310	3570	n.d.	585	13.8	-	-
AYW-I	43	1,014	23,753	69.4	28.3	7.1	982	1140	370	4780	450	1.83	4330	840	3330	n.d.	948	10.1	-	-
Effluent of WWTP																				
GAW-E	32	44	1,375	14.1	26.1	7.2	548	930	300	2450	400	1.48	2130	610	1350	390	162	19.7	-	-
WSW-E	27	337	12,325	44.1	26.3	6.6	471	800	220	2110	330	1.36	1870	410	900	740	201	18.2	-	-
TAW-E	71	1,776	25,185	230	27.8	6.6	505	820	250	2110	390	1.51	2060	320	1240	700	1716	8.1	-	-
JNW-E	112	3,641	32,580	405	26.9	7.2	745	770	280	2460	480	1.31	2140	280	4150	n.d.	519	13.4	-	-
AYW-E	43	1,014	23,753	69.4	26.2	7.0	788	1040	340	4200	460	1.53	4030	740	1600	340	722	11.6	-	-
GPW-E	60	1,701	28,373	173	27.8	6.7	596	910	280	2830	380	1.30	2460	570	1270	650	399	16.1	-	-
	Primary activated sludge (PAS)							12221	3093	2138	4982	45.0	-	-	-	-	7.80	-0.4	0.6	2
	Waste activated sludge (WAS)							6547	2950	1518	3820	83.6	-	-	-	-	6.15	-0.3	0.3	2
WWTP[f]	Sludge from drying treatment system							14413	5227	1942	4942	107	-	-	-	-	7.85	1.7	0.0	2
	Supernatant fluid from PAS							95.4	21.2	61.0	33.7	0.49	-	-	-	-	0.01	8.1	0.3	2
	Supernatant fluid from WAS							25.7	15.1	56.0	37.3	0.09	-	-	-	-	0.01	12.8	0.3	2

n.d.: not detected

[a] GAW: Gyeongan, WSW: Wangsuk, TAW: Tan, JNW: Jungnang, AYW: Anyang, and GPW: Gulpo

[b] data from the Ministry of Environment (http://eng.me.go.kr)

[c] calculated as dividing population by drainage area

[d] HCO_3 ≈ Total alkalinity

[e] standard error

[f] see Supplementary Figure 1 and the text for more details; Concentration units in ppm (d.w.)

Supplementary Table 4

Li isotope geochemistry of Seoul tap water samples.

Sample	Li	δ^7Li
	nM	(‰)
T1	40.8	26.9
T2	50.4	25.3
T3	55.1	23.7
T4	103	23.3

Supplementary Table 5

Elemental and Li isotope geochemistry of Li-rich materials in this study.

Sample		Ca	Mg	Na	K	Sr	Li	δ^7Li	$1\sigma_m$[a]	n
		(ppm d.w.)						(‰)		
Therapeutic drug (TD)	Lithium carbonate from Myung In Pharm	15.0	367	1,569	92.7	0.02	108,743	12.2	0.1	4
	Lithium carbonate from Whan In Pharm	906	394	608	124	1.35	100,734	13.3	0.3	2
Food waste treatment plant (FWTP)	Compost	24,957	1,256	8,080	8,423	65.5	0.39	9.3	-	1
	Wastewater before treatment	1,087	184	2,416	2,122	3.44	0.02	18.3	0.5	2
	Wastewater after treatment	903	162	6,818	1,854	2.82	0.02	20.4	-	1
Secondary Li-ion Battery (LIB)	$LiFePO_4$ (Commercial, China)	147	n.d.	2,286	903	5.43	40,592	8.5	0.0	2
	$LiFePO_4$/NC[b] (Korea)	106	n.d.	108	175	0.83	45,823	2.4	0.0	2
	$LiFePO_4$/C (Korea)	14.2	n.d.	57.3	52.6	0.13	74,425	3.1	0.1	2
	$LiCoO_2$ (Commercial, Korea)	123	2,640	127	50.3	4.50	76,129	12.6	0.0	2
Detergent	SuperTie	18,022	900	216,069	1,304	15.0	1.39	8.0	0.5	2
	TECH	26,845	1,484	280,105	334	22.1	2.92	18.8	0.7	2
	BEAT	16,208	1,639	261,328	385	11.7	0.53	13.2	0.2	2
	Spark	15,909	844	272,990	372	11.4	1.38	21.4	0.9	3

n.d.: not detected
[a] standard error
[b] NC: Nitrogen-doped Carbon

Supplementary Note

The geology of the Han River (HR) basin consists of Precambrian gneiss, Mesozoic granites, and Paleozoic carbonates[1-3]. The Bukhan River (BR) drains Precambrian gneiss and Mesozoic granites, while the upper Namhan River (NR) flows through Paleozoic carbonates/clastic sediments and then the lower NR drains Precambrian gneiss and Mesozoic silicates. Neither salt-bearing rocks nor evaporates occur in the HR basin[4]. The land occupation of the HR basin is mainly forested (46.2%), followed by residential and business areas (33.8%) and agricultural lands (11.1%) (Supplementary Table 1). The mean annual temperature is 13.1°C, ranging from 8.1°C in the winter to 18.7°C in the summer[5]. The mean precipitation is 1450 mm/year, of which approximately two-thirds occurs between June and September[5].

Supplementary References

1. Ryu, J.-S., Lee, K.-S. & Chang, H.-W. Hydrogeochemical and isotopic investigations of the Han River basin, South Korea. *J Hydrol*. **345**, 50-60 (2007).
2. Ryu, J.-S., Lee, K.-S., Chang, H.-W. & Shin, H. S. Chemical weathering of carbonates and silicates in the Han River basin, South Korea. *Chem. Geol*. **247**, 66-80 (2008).
3. Ryu, J.-S., Lee, K.-S., Chang, H.-W. & Cheong, C-S. Uranium isotopes as a tracer of sources of dissolved solutes in the Han River, South Korea. *Chem. Geol*. **258**, 354-361 (2009).
4. Chough, S. K., Kwon, S. T., Ree, J. H. & Choi, D. K. Tectonic and sedimentary evolution of the Korean peninsula: a review and new view. *Earth-Sci. Rev*. **52**, 175–235 (2000).
5. KMA, 2017. Annual Climatological Report. Korea Meteological Administration.
